# Three‐year‐old with severe‐combined immunodeficiency and a mildly expansile T2/FLAIR signal abnormality

**DOI:** 10.1111/bpa.13173

**Published:** 2023-06-05

**Authors:** Vanessa L. Smith, William T. Harrison

**Affiliations:** ^1^ Department of Pathology Duke University Medical Center Durham North Carolina USA; ^2^ Present address: Department of Pathology Wake Forest Baptist Health Winston Salem North Carolina USA

**Keywords:** PML, progressive multifocal leukoencephalopathy, severe combined immunodeficiency

BOX 1 **Virtual glass slide**

Access at https://isn‐slidearchive.org/?col=ISN&fol=Archive&file=BPA‐22‐08‐197.svs

## 1 CLINICAL HISTORY

**FIGURE 1:**
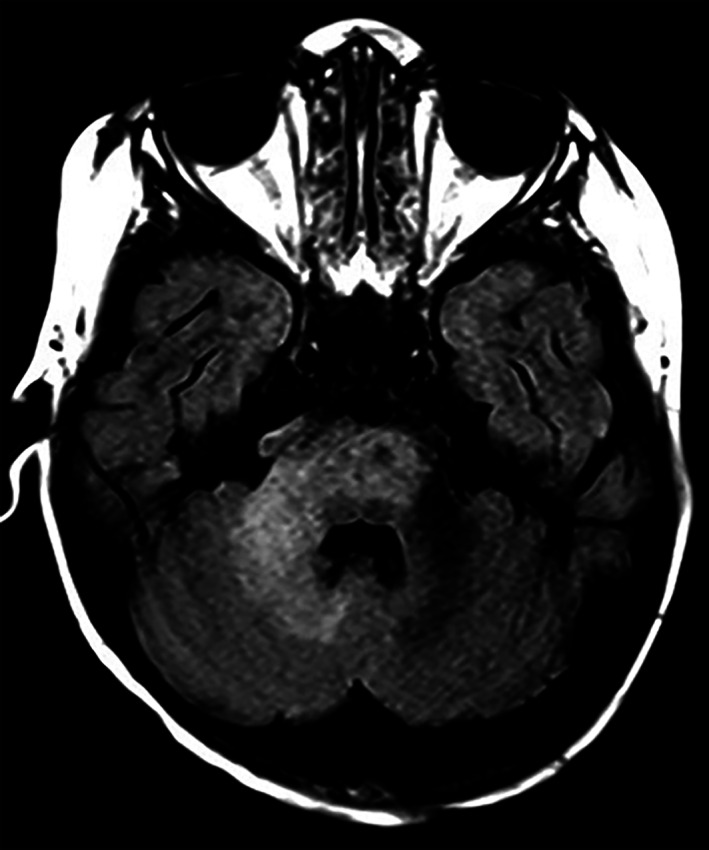
FLAIR MRI appearance. A mildly expansile T2 signal abnormality within the bilateral pons is present with extension into the right cerebellar white matter and medulla with sparing of the gray matter. No associated enhancement or diffusion restriction was present.

A 3‐year‐old male presented with fever and respiratory distress. He had a history of T‐cell negative, B‐cell positive, NK‐cell positive severe combined immunodeficiency (SCID); he was status post failed allogeneic bone marrow transplant and subsequent matched‐umbilical cord blood transplantation with graft versus host disease and multiple chronic infections. Upon admission, a chest X‐ray showed diffuse patchy opacities; blood and sputum cultures and viral panels were unrevealing. He started broad spectrum antibiotic therapy and received oseltamivir for possible influenza. He was already on voriconazole therapy secondary to candida sinusitis. After a protracted course with limited improvement, his mental/physical status acutely worsened. Brain magnetic resonance imaging (MRI) showed a mildly expansile T2/FLAIR signal abnormality within the white matter of the pons, medulla, and right cerebellar hemisphere (Figure 1). Given the patient's poor prognosis, family made the decision to withdraw care. A complete autopsy was done.

## 2 FINDINGS

**FIGURE 2:**
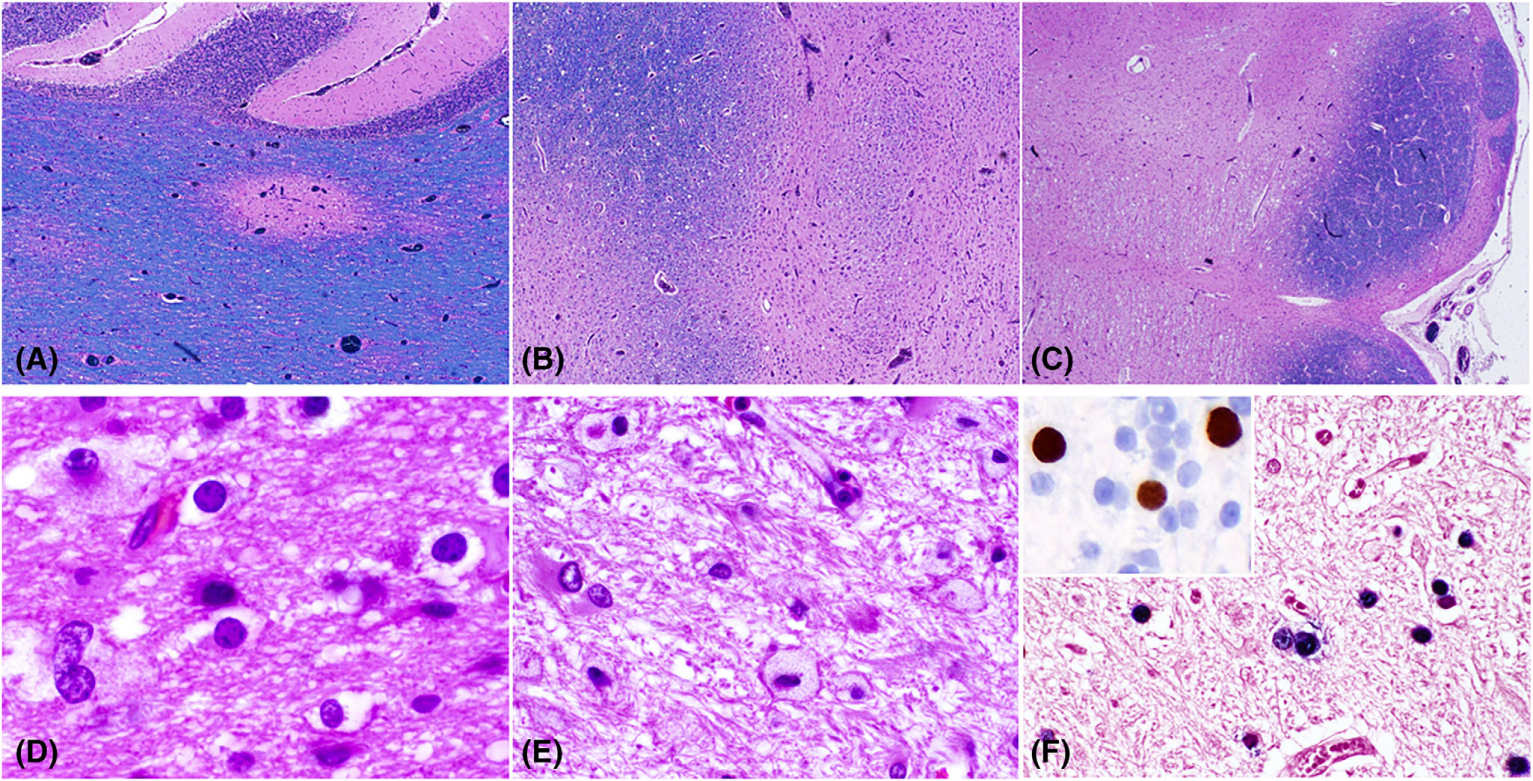
PML appearance in the cerebellum, pons, and medulla. PML plaques can be appreciated at low magnification on Luxol Fast Blue in the cerebellum (A), pons (B), and medulla (C). Bizarre astrocytes, oligodendrocytes with viral cytologic atypia, and macrophages are seen on H&E (D, E). Infected cells are positive for JCV CISH (F) and stain with SV40 (inset, F). Objective magnifications: 2× (C), 4× (A, B), 20× (F inset), 40× (E, F), 60× (D).

Sections of the pons show multiple, focally confluent foci of demyelination (Box 1; Figure 2). Axons are intact in the smaller foci. In larger areas of demyelination, macrophages are prominent and there is necrosis. Oligodendrocytes at the periphery of plaques have large homogenous nuclei. There are numerous enlarged reactive astrocytes with bizarre hyperchromatic nuclei and abundant eosinophilic cytoplasm. Sections of cerebellum and medulla are similar (Figure 2).

## 3 DIAGNOSIS

Progressive multifocal leukoencephalopathy (PML).

## 4 DISCUSSION

The patient had chronic John Cunningham virus (JCV) infection since his initial bone marrow transplant but had been stable. Cerebrospinal fluid JCV qPCR revealed 2,400,000 copies/mL 3 days before care withdrawal. On autopsy, SV40 immunostain was positive in the pons, medulla, and cerebellum. JCV in situ hybridization (ISH) performed on medulla and cerebellum was also positive.

PML is a debilitating and often fatal central nervous system demyelinating disorder caused by JCV [1]. JCV was first described in 1971. Initially, PML was thought to be a rare complication of hematologic cancers. Since the AIDS epidemic in the 1980s, PML has been associated primarily with HIV [1]. In 2005, natalizumab treatment was identified as a risk factor for PML in multiple sclerosis [1]. Though PML can affect immunocompromised individuals with a wide range of etiologies for their status, little has been published on those with immunodeficiencies such as SCID.

JCV is in the polyoma virus family and is thought to spread via multiple modes that center on viral entry through the oropharynx. Persistent latent infection establishes in the genitourinary tract, bone marrow, and lymphoid tissue. Neurotropic JCV is rearranged from the wildtype virus to include more transcription factor binding sites, conferring a growth advantage [1]. Immune compromise is permissive of active neurotropic infection.

PML presents in patients as changes in personality, cognition, motor deficits, gait difficulties, homonymous hemianopia, and rarely seizures [1]. Our patient presented with 1 month of night terrors at 23 months of age.

Lesions are typically found in frontal or parieto‐occipital locations and cerebellar peduncles, but are also found in the basal ganglia and thalamus [1]. On computed tomography imaging, lesions appear hypointense within white matter. On MRI, lesions appear hyperintense on T2 and FLAIR and hypointense on T1 [1].

On gross exam, PML is characterized by multifocal demyelination. Early stage lesions are small, ovoid, lesions maximal near the cortical gray‐white matter junction. Intermediate stage lesions are coalescent. Late stage PML has large depressed lesions in white matter [1].

On microscopic exam, PML infection demonstrates oligodendrocytes harboring viral inclusions, bizarre astrocytes, and demyelination [1]. Axonal injury, macrophage and inflammatory infiltrate, and, rarely, neuronal infection can be seen. Infected oligodendrocytes have enlarged nuclei ranging from round with violaceous nuclear inclusions to central clearing and marginated chromatin. Bizarre astrocytes are present throughout demyelination plaques and are frequently multinuclear with atypical hyperchromatic nuclei and pleomorphic cytoplasm. Macrophages are dispersed throughout areas of active myelin breakdown. They are perivascular in later stages [1, 2]. Virions can be found on electron microscopic exam and virally infected cells are often immunohistochemically positive for Ki‐67, p53 and SV40 or JCV [1, 2]. JCV ISH is another method by which JC virus can be detected in formalin‐fixed tissues [2]. JCV ISH probes may cross‐react with BK virus while SV40 immunostain cross‐reacts with all polyomaviruses [3]. Previous studies have determined that JCV ISH is more specific for detecting JCV. SV40 immunostain is more sensitive for detecting JCV [2].

## AUTHOR CONTRIBUTIONS

Vanessa L. Smith wrote the original draft. William T. Harrison reviewed and edited the draft.

## CONFLICT OF INTEREST STATEMENT

The authors declare no conflict of interest.

## ETHICS STATEMENT

All data related to this case are deidentified.

## Data Availability

Data sharing is not applicable to this article as no new data were created or analyzed in this study.

## References

[1] Cortese I , Reich DS , Nath A . Progressive multifocal leukoencephalopathy and the spectrum of JC virus‐related disease. Nat Rev Neurol. 2021;17(1):37–51. 33219338 10.1038/s41582-020-00427-y PMC7678594

[2] Munoz‐Marmol AM , Mola G , Fernandez‐Vasalo A , Vela E , Mate JL , Ariza A . JC virus early protein detection by immunohistochemistry in progressive multifocal leukoencephalopathy: a comparative study with in situ hybridization and polymerase chain reaction. J Neuropathol Exp Neurol. 2004;63(11):1124–30. 15581180 10.1093/jnen/63.11.1124

[3] Fritzsche FR , Pianca S , Gaspert A , Varga Z , Wang L , Farrell MP , et al. Silver‐enhanced in situ hybridization for detection of polyomavirus DNA in patients with BK virus nephropathy. Diagn Mol Pathol. 2011;20(2):105–10. 21532490 10.1097/PDM.0b013e3182015074

